# When the genome bluffs: a tandem duplication event during generation of a novel *Agmo* knockout mouse model fools routine genotyping

**DOI:** 10.1186/s13578-021-00566-9

**Published:** 2021-03-16

**Authors:** Sabrina Sailer, Stefan Coassin, Katharina Lackner, Caroline Fischer, Eileen McNeill, Gertraud Streiter, Christian Kremser, Manuel Maglione, Catherine M. Green, Daniela Moralli, Alexander R. Moschen, Markus A. Keller, Georg Golderer, Gabriele Werner-Felmayer, Irmgard Tegeder, Keith M. Channon, Benjamin Davies, Ernst R. Werner, Katrin Watschinger

**Affiliations:** 1 Institute of Biological Chemistry, Biocenter, Medical University of Innsbruck, Innsbruck, Austria; 2 Institute of Genetic Epidemiology, Department of Genetics and Pharmacology, Medical University of Innsbruck, Innsbruck, Austria; 3 Institute of Clinical Pharmacology of the Medical Faculty, Goethe-University, Frankfurt (Main), Germany; 4 Wellcome Centre for Human Genetics, University of Oxford, Oxford, United Kingdom; 5 Division of Cardiovascular Medicine, British Heart Foundation Centre of Research Excellence, University of Oxford, Oxford, United Kingdom; 6 Department of Radiology, Medical University of Innsbruck, Innsbruck, Austria; 7 Department of Visceral, Transplant and Thoracic Surgery, Medical University of Innsbruck, Innsbruck, Austria; 8 Department of Internal Medicine I, Gastroenterology, Endocrinology and Metabolism, Medical University of Innsbruck, Innsbruck, Austria; 9 Institute of Human Genetics, Medical University of Innsbruck, Innsbruck, Austria; 10 Institute of Biological Chemistry, Biocenter, Medical University of Innsbruck, Innrain 80, 6020 Innsbruck, Austria

**Keywords:** Homologous recombination, Mouse models, Ether lipid metabolism, Genomic structural variation, Alkylglycerol monooxygenase

## Abstract

**Background:**

Genome editing in mice using either classical approaches like homologous recombination or CRISPR/Cas9 has been reported to harbor off target effects (insertion/deletion, frame shifts or gene segment duplications) that lead to mutations not only in close proximity to the target site but also outside. Only the genomes of few engineered mouse strains have been sequenced. Since the role of the ether-lipid cleaving enzyme alkylglycerol monooxygenase (AGMO) in physiology and pathophysiology remains enigmatic, we created a knockout mouse model for AGMO using EUCOMM stem cells but unforeseen genotyping issues that did not agree with Mendelian distribution and enzyme activity data prompted an in-depth genomic validation of the mouse model.

**Results:**

We report a gene segment tandem duplication event that occurred during the generation of an *Agmo* knockout-first allele by homologous recombination. Only low homology was seen between the breakpoints. While a single copy of the recombinant 18 kb cassette was integrated correctly around exon 2 of the *Agmo* gene, whole genome nanopore sequencing revealed a 94 kb duplication in the *Agmo* locus that contains *Agmo* wild-type exons 1–3. The duplication fooled genotyping by routine PCR, but could be resolved using qPCR-based genotyping, targeted locus amplification sequencing and nanopore sequencing. Despite this event, this *Agmo* knockout mouse model lacks AGMO enzyme activity and can therefore be used to study its physiological role.

**Conclusions:**

A duplication event occurred at the exact locus of the homologous recombination and was not detected by conventional quality control filters such as FISH or long-range PCR over the recombination sites. Nanopore sequencing provides a cost convenient method to detect such underrated off-target effects, suggesting its use for additional quality assessment of gene editing in mice and also other model organisms.

## Background

Gene function studies require *in vivo* investigations to pin down the exact roles in higher ordered systems. To mirror human disorders in model organisms, mice are frequently used because they often reflect the disease when the responsible gene is modified [1]. The first transgenic mice were created in 1980 by DNA microinjections into fertilized eggs [2]. This method was soon replaced by another approach using gene targeting by homologous recombination in mouse embryonic stem cells [3]. Shortly after this milestone, the Cre/loxP system was added for somatic mutations to study tissue specific effects [4]. Homologous recombination was also used by the EUCOMM consortium to generate stem cells with targeted mutations of many protein-coding genes [5]. Most recent efforts to generate mouse models comprise genome editing of mouse embryonic stem cells by CRISPR/Cas9 application [6] and methods to reduce and detect off-target effects of CRISPR/Cas9 [7, 8]. However, significant on-target mutagenesis, such as large deletions and more complex genomic rearrangements at the targeted sites, has been recently reported to accompany CRISPR/Cas9 editing [9]. Genetic characterization of 40 transgenic mouse lines demonstrated that large deletions and structural variations frequently occurring at the integration sites might interfere with the obtained results and would require careful selection of controls [10].

Alkylglycerol monooxygenase (AGMO, E.C. 1.14.16.5) was considered an orphan enzyme (an enzyme with unknown coding gene) until 2010, when we assigned it to the gene *Tmem195* [11]. The physiological function of AGMO is slowly emerging [12]. In *C. elegans*, AGMO (BE10.2) was suggested to be a potential candidate in daf-2 mediated longevity [13]. Additionally, AGMO might play a role in inflammation [14]. Mutations in the *AGMO* gene were found to be associated with visceral leishmania relapse in humans [15], but other genes might also be involved [16]. A role of AGMO in platelet-activating factor regulation was suggested [17], but could not be reproduced in our cell system [14]. Additionally, AGMO has been associated with microcephaly [18], autism [19], energy homeostasis [20] and congenital heart disease [21] in humans. Mouse models lacking all ether lipids due to knockout of enzymes early in the biosynthetic pathway showed that these lipids are crucial for male fertility, protection from cataract and brain fine structure development [22]. AGMO is the only enzyme capable of cleaving the ether bond of alkylgylcerols and lyso-alkylglycerophospholipid species. The effects of defective ether lipids degradation on metabolism have not been shown yet.

Therefore, we generated an *Agmo* knockout mouse using EUCOMM stem cells containing the Cre/loxP system. While the inactivation of AGMO enzymatic activity in these knockout mice was achieved as expected, difficulties in genotyping the animals by conventional PCR led us to a detailed analysis of the genome by nanopore sequencing. This revealed a duplication of a 94 kb segment of the *Agmo* wild-type gene that occurred at the locus of homologous recombination.

## Results

### Generation and validation of ***Agmo***-deficient mice

**Fig. 1:**
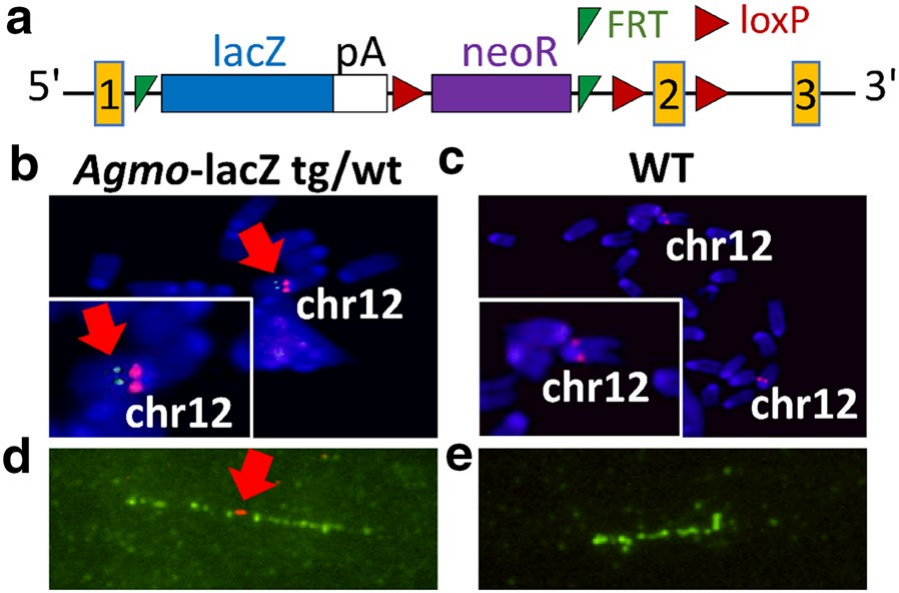
*Agmo* knockout-first allele construct and confirmation of cassette integration in the *Agmo* region of the mouse genome. **a** EUCOMM construct for *Agmo* knockout-first (*Agmo*-lacZ) allele. The critical exon 2 is flanked by loxP sites to create a floxed allele for conditional knockout. pA: polyadenylation sequence; FRT: FLP recognition target. **b**, **c** FISH experiments in heterozygous *Agmo* knockout (*Agmo*-lacZ tg/wt, **b**) and wild-type (WT, **c**) fibroblasts using BAC probes against chromosome 12 (red) and the cassette (green, red arrow). Insets show a 2x magnification of the region of interest. Fiber-FISH experiments show integration of the cassette (red signal, indicated by the red arrow) in the middle of the *Agmo* locus (green signal) in heterozygous cells (**d**). Wild-type fibers (**e**) showed no signal for the cassette

We generated *Agmo* knockout-first mice (*Agmo*-lacZ) using EUCOMM stem cells harboring a transgenic cassette with conditional potential (Fig. 1a). Two different EUCOMM clones were purchased but only one was successfully expanded and yielded chimeric animals by blastocyst injection, which then transmitted the targeted allele through the germline. Integration was validated by long-range PCR over the 5’ and 3’ integration sites of homologous recombination (see Additional file 1: Fig. S1 for a schematic drawing of all genotyping primer positions and the primer positions for long-range PCR and synthesis of the FISH probe) in both, the stem cells and mice (Additional file 1: Fig. S2) and sequencing of the integration region of the products. Presence of the distal loxP site was also confirmed by PCR and sequencing. Integration of the transgenic cassette on one of the two chromosomes 12 was detected by fluorescence in situ hybridization (FISH) in fibroblasts from a heterozygous animal, using a probe specific for chromosome 12 and a second probe specific for the transgene cassette (4,982 bp, Fig. 1b). No cassette signal was obtained in wild-type fibroblasts (Fig. 1c). Fiber-FISH experiments on extended chromatin fibers from the same fibroblasts showed that 48 % of the signals of BAC RP24-270A18 spanning the *Agmo* locus co-localized with the cassette probe (Fig. 1d red arrow) while in wild-type fibroblasts none of the fibers showed a signal for the cassette (Fig. 1e).

### Characterization of ***Agmo***-deficient mice

**Fig. 2:**
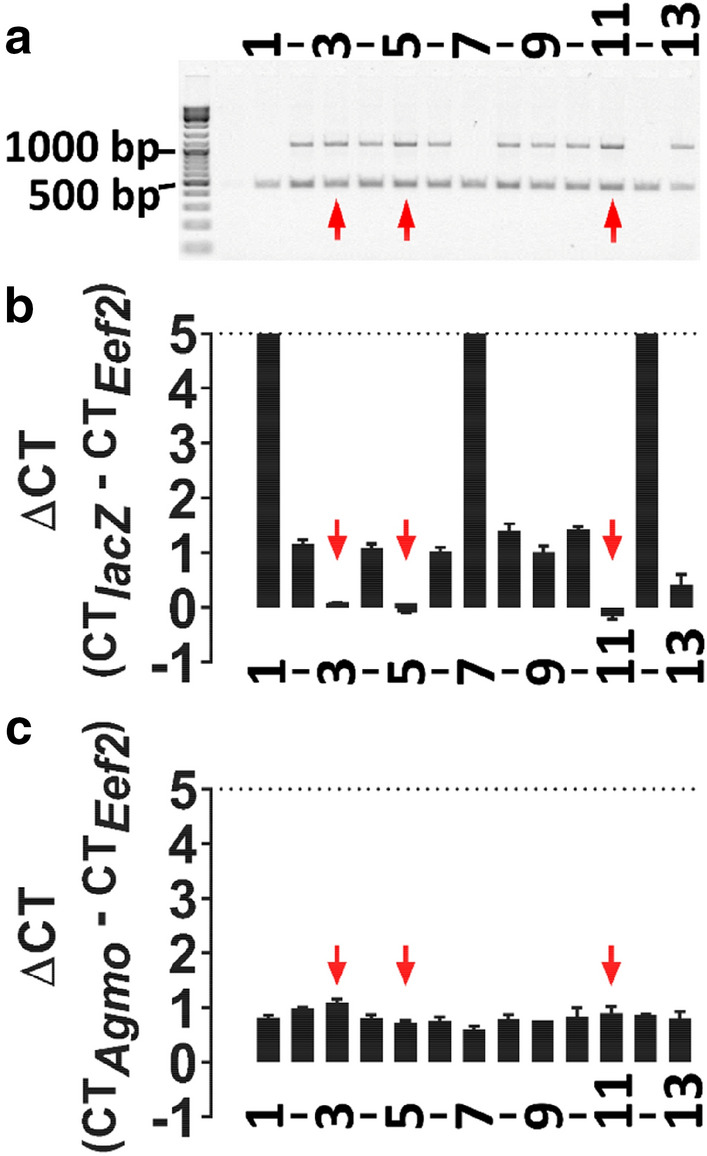
Genotyping of *Agmo* transgenic mice by qPCR and by conventional PCR. **a** Conventional genotyping of *Agmo*-lacZ cohorts (wt/wt: 476 bp, tg/wt: 476 bp + 1090 bp). **b** *LacZ* qPCR to count allele copy numbers of the *Agmo*-lacZ transgene and **c** *Agmo*-WT qPCR to determine copy numbers of the native *Agmo* allele. Red arrows indicate homozygous *Agmo*-lacZ animals determined by *lacZ* qPCR. Y-axes were cut off at ∆CT 5. Data shown as mean ± S.D.

We set up heterozygous breeding pairs and genotyped litters for the knockout construct, but were not able to identify homozygous knockout animals (1090 bp genotyping PCR band only, absence of the 476 bp wild-type band, Fig. 2a). Therefore, we hypothesized that loss of *Agmo* would lead to embryonic lethality. AGMO is a tetrahydrobiopterin (BH4)-dependent enzyme and embryos of mouse models deficient for *Gch1*, the rate-limiting enzyme in BH4 biosynthesis, die at embryonic day 11.5 (E-11.5) [23]. We prepared embryos at E-12.5 after timed mating of heterozygous animals and genotyped 50 embryos in total. Twelve embryos resulted as wild-type (24 %) and 38 embryos as heterozygous for the transgenic cassette (76 %). These results gave a first hint that the genotyping method employed might be unable to discriminate between heterozygous and homozygous animals, since Mendelian inheritance of the *Agmo*-lacZ allele was expected to be 25 % (wild-type): 50 % (heterozygous): 25 % (homozygous) rather than indicating embryonic lethality which would result in 33 % wild-type and 67 % heterozygous animals. We prepared mouse embryonic fibroblasts (MEFs) of selected litters to exclude contamination with material from the heterozygous mother but also here a wild-type PCR band was always detected. We next ruled out PCR contamination by rigorous spatial separation of genomic DNA (gDNA) preparation and PCR reactions and by repetition of the genotyping PCR in another laboratory with fresh reagents.

To tackle the potential ambiguities of the PCR results, we established genotyping by qPCR allowing us to quantify alleles harboring (i) the lacZ cassette for *Agmo*-lacZ mice (Fig. 2b), (ii) the recombinant region including the loxP site around exon 2 for *Agmo*-flox mice (litters of *Agmo*-lacZ mice bred with FlpE deleter mice) and (iii) the wild-type gene (Fig. 2c), all in relation to eukaryotic translation elongation factor 2 (*Eef2)* as reference gene. All genotypes were determined by calculating the difference of ∆CT values between the different mice in the mutant qPCR reactions (∆∆CT of approximately 1 between homozygous and heterozygous transgenic animals). Allele quantification by qPCR showed the expected Mendelian inheritance ratios (Additional file 1: Table S1) for the different mutant alleles (Fig. 2b), despite the unchanged presence of the same amount of wild-type allele amplification in conventional (Fig. 2a) and qPCR experiments (Fig. 2c). Homozygous animals displaying a pattern consistent with a heterozygous genotyping using the conventional PCR are indicated by red arrows (Fig. 2). Thus, we pursued genotyping only by qPCR of all *Agmo* mice, as also others have suggested for testing zygosity in transgenic laboratory animals [24] and for quality control to assure correct integration number of the transgenic cassette [25].

### Determination of AGMO activity in ***Agmo***-lacZ and ***Agmo***-Δexon2 animals

**Fig. 3:**
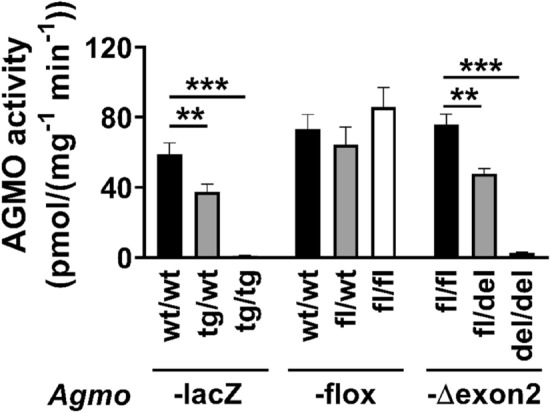
AGMO enzyme activities in livers of *Agmo*-lacZ, *Agmo-*Δexon2 and *Agmo*-flox mice as compared to littermate controls. AGMO enzyme activities were determined in livers from 8–12 weeks old transgenic mice of *Agmo*-lacZ (n = 12–20), *Agmo*-flox (n = 16–18) and *Agmo*-Δexon2 mice (n = 8–9). Data summarizes activities in male and female mice. (p < 0.01 **; p < 0.001 ***). Data shown as mean ± S.E.M.

Next, we pursued a second independent approach to confirm the results found by qPCR. We analyzed enzymatic activities of AGMO in 11 organs from wild-type, heterozygous or homozygous animals. Liver activities pooled for male and female mice are shown in Fig. 3. A decline in enzyme activities coincided with the corresponding genotype. Heterozygous *Agmo*-lacZ animals (grey bars) had a clearly reduced enzyme activity (p = 0.0026) and homozygous *Agmo*-lacZ animals (white bars) had AGMO activities less than 2 % (p < 0.0001) when compared to wild-type controls (black bars). No difference was found between male and female mice. Additional file 1: Fig. S3A shows all 11 tested organs of male and female mice separately. We also analyzed AGMO activities in *Agmo*-flox mice and – as expected for animals with floxed alleles – observed no alterations in liver activities in all three genotypes (Fig. 3).

Using the *Agmo*-flox mice we then created a knockout mouse strain lacking exon 2 (*Agmo*-Δexon2) by crossing them with a CMV-cre total deleter mouse strain in which the Cre recombinase is active already in germ lines. AGMO activities in the liver of homozygous and heterozygous mice from this *Agmo*-Δexon2 strain followed the same pattern as those in *Agmo*-lacZ animals (Fig. 3; Additional file 1: Fig. S3B shows all 11 organs).

In parallel, gene expression patterns of endogenous *Agmo* gene were determined in male and female *Agmo*-lacZ animals by RT-qPCR (see Additional file 1: Fig. S4). AGMO activity and gene expression correlated significantly in both wild-type (p = 0.03 for females and p < 0.0001 for males) and heterozygous animals (p < 0.0001 for both females and males).

### Phenotyping and expression pattern analysis of *Agmo*-lacZ and *Agmo* -∆exon2 animals

**Fig. 4:**
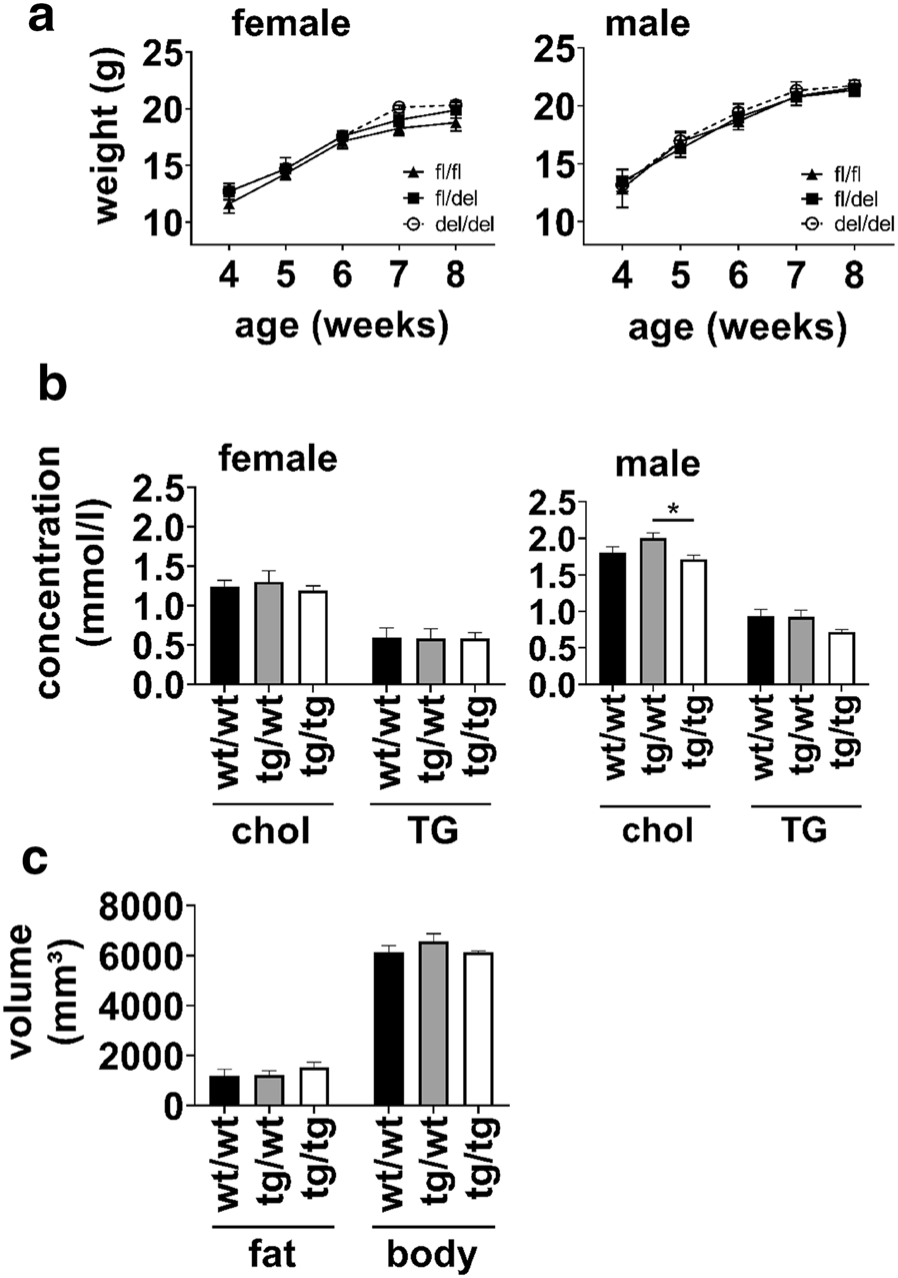
Phenotypic characterization of *Agmo*-lacZ and *Agmo*-Δexon2 mice. **a** Weight gain from 4 to 8 weeks was determined in *Agmo*-Δexon2 mice (male mice: n = 3–11; female mice: n = 6–9). Open circles, dashed line: homozygous *Agmo*-Δexon2 mice (del/del); solid squares, solid line: heterozygous *Agmo*-Δexon2 mice (fl/del); solid triangles, solid line: floxed control litter mates (fl/fl). **b** Measurement of cholesterol (chol) and triglycerides (TG) in plasma samples from *Agmo*-lacZ male and female mice (n = 4–7). **c** Body fat quantification by MRI in male *Agmo*-lacZ mice (n = 4). (p < 0.05 *) Data shown as mean ± S.E.M.

Under normal housing conditions, homozygous *Agmo*-lacZ and *Agmo*-∆exon2 mice had no obvious phenotypic changes compared to their wild-type littermates. This included also color, activity, weight and milk spot in newborn pups as well as grooming activities of their mothers. Furthermore, weight gain of weaned litters from *Agmo*-∆exon2 animals was monitored weekly from age four up to eight weeks (Fig. 4a) and also here, no significant changes could be detected. Plasma cholesterol and triglycerides were measured and were unchanged between wild-type, heterozygous and homozygous *Agmo*-lacZ female (left panel) and male (right panel) animals except for a significant change in cholesterol between heterozygous (2.00 ± 0.07 mmol/l) and homozygous male mice (1.71 ± 0.06 mmol/l, p = 0.02) which was however not significant for wild-type (1.81 ± 0.08 mmol/l, p = 0.6) (Fig. 4b). Additionally, we analyzed body fat of these animals by magnetic resonance imaging (MRI) (Fig. 4c). Again, no changes were detected between all three genotypes. As quality control of semi-automated MRI analysis we performed Pearson correlation analysis of body weight (g) versus body volume (mm³), calculated from T2-weighted water-separated images and found a strong direct correlation between both values (r = 0.87, p = 0.0003).

One of the advantages of the EUCOMM knockout-first allele is that mice harboring such an allele can be used as reporter for expression studies of the gene of interest. We could successfully perform lacZ stainings in a set of organs in *Agmo*-lacZ mice (Additional file 1: Fig. S5, S6, S7). In the liver, *Agmo-lacZ* expression was observed in hepatocytes close to the central vein and in von Willebrand Factor immunoreactive endothelial cells, but was not found in F4/80 positive Kupffer cells (Additional file 1: Fig. S5). In the intestine, *lacZ* expression was found in basal epithelial cells and gastric glands, and it reached up to the luminal epithelium in the colon (Additional file 1: Fig. S6). In testis, *lacZ* expression was predominantly found in DDX4/VASA positive testicular germ cells (Additional file 1: Fig. S7), but was not observed in SOX9 positive Sertoli cells.

### Characterization of wild‐type insertion by long‐range PCR and targeted locus amplification (TLA)

Our results from both conventional and qPCR genotyping experiments indicated presence of a segment of *Agmo* wild-type allele that did not interfere with successful functional knockout of AGMO activity. By long-range PCRs with one primer binding in the wild-type region which should be missing in a correct homozygous *Agmo*-lacZ mouse and the other primer located at increasing distance in the surrounding context sequence we observed that this integrated wild-type sequence was at least 25 kb long.

We used Targeted Locus Amplification (TLA) to map the location of the transgene and the wild-type segment. TLA applies the principle of proximity ligation to sequence the context of a target locus [10, 26]. We selected two probe sets, one for the lacZ cassette and one for the wild-type segment (expected to disappear in *Agmo*-lacZ mice). Results with the probe set for the lacZ cassette confirmed integration exclusively in chromosome 12 in the expected region (Additional file 1: Fig. S8A, S9A). With the second probe set, a wild-type segment in close proximity to the lacZ cassette could be detected (Additional file 1: Fig. S8B, S9B). Importantly, no integration in other genomic sites was observed (Additional file 1: Fig. S8B, S9B).

### Duplication mapping by nanopore sequencing

We used whole genome nanopore sequencing to refine the structure of the assumed duplication. Sequencing generated about 13 Gb from a single flow cell with an N50 value of 40.7 kb (Additional file 1: Table S2), providing a mean genome coverage of 4.37X for minimap2 alignment and 4.15X for alignment using NGMLR. Coverage at the *Agmo* region with homology to the targeting vector was 8-15X as judged by visual inspection of the minimap2 alignment.

**Fig. 5:**
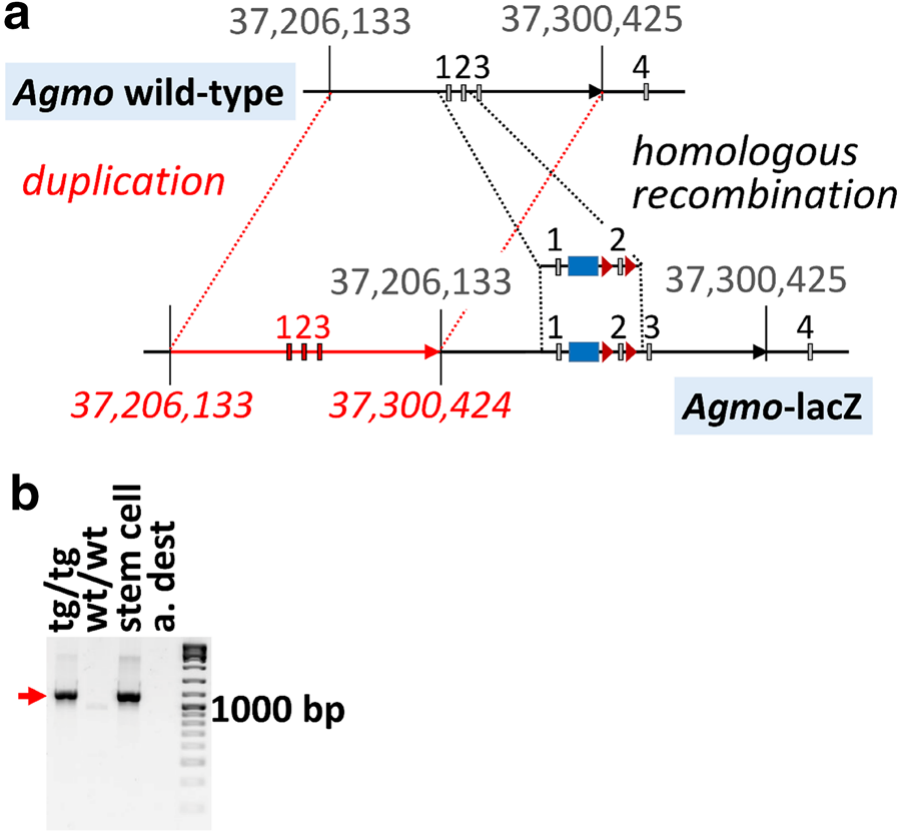
A duplication event of wild-type DNA during homologous recombination of the L1L2_Bact_P cassette (see Fig. 1a for details) into the *Agmo* locus. **a** Schematic drawing of the homologous recombination event of the transgenic cassette into the wild-type Agmo locus (indicated by the dashed grey line) and the tandem duplication event of a 94 kb *Agmo* wild-type segment inserting unmodified exons 1–3 of the *Agmo* locus upstream of the cassette (indicated by the dashed red lines). The numbers refer to chromosome 12 of the mouse, assembly GRCm38.p6 accession NC_000078.6. A potential mechanism leading to this duplication event is outlined inDiscussion section. **b** PCR across the duplication breakpoint (see Additional file 1: Fig. S10) in homozygous *Agmo*-lacZ animals (tg/tg), stem cells used for generation of the *Agmo*-lacZ mouse and in wild-type mice (wt/wt)

All mapping and structural variant (SV) detection algorithm combinations concordantly reported a 94 kb tandem duplication of an *Agmo* wild-type fragment encompassing the first three exons (Fig. 5a). They detected the same downstream breakpoint and only minor variance in localization of the upstream breakpoint was proposed (chr12:37,206,133–37,300,425 using minimap2 + sniffles, chr12:37,206,699–37,300,425 using NGMLR + sniffles, chr12:37,206,133–37,300,424 using minimap2 + SVIM and chr12:37,206,132 or chr12:37,206,735–37,300,424 using NGMLR + SVIM; all numbers refer to assembly GRCm38.p6). The detected tandem duplication was the only duplication on chromosome 12 originating in the broader *Agmo* region as well as the best-supported duplication on chromosome 12 in both the sniffles and the SVIM data. Manual inspection of 8 selected reads supporting the structural variation (Additional file 2) consistently showed chr12:37,206,133 as breakpoint (Additional file 1: Fig. S10). Position chr12:37,206,133 is localized within an L1 long interspersed nuclear element (LINE), while no remarkable sequence elements were found at chr12:37,300,424 and only low homology was present between the regions (Additional file 1: Fig. S11).

The breakpoint was confirmed using PCR with an upstream-primer starting at chr12:37,299,719 against a downstream primer starting at chr12:37,206,553, which would raise an amplicon only if the duplication is present as suggested by the nanopore data. This indeed yielded the 1,127 bp product only in homozygous *Agmo*-lacZ animals and in the stem cell clone used to generate the mouse, but not in wild-type animals (Fig. 5b) with a sequence perfectly matching the model shown in Fig. 5a (sequences given in Additional file 2).

#### Assessment of AGMO mRNA in transgene animals

To assess the impact of this insertion on the sequence of the mRNA, we sequenced RT-PCR products spanning the complete reading frame of cDNAs prepared from liver RNAs of wild-type, homozygous *Agmo*-flox, and homozygous *Agmo*-Δexon2 animals (four cloned PCR products each for three animals of each genotype). The results showed that exon 3 was never followed by another set of exons 1, 2, 3 but always by exon 4, indicating that the additional exons 1, 2, 3 were not included into the mature mRNA upon transcription of the modified *Agmo* locus.

## Discussion

Various pitfalls in the generation of transgenic animal models, such as genetic background, flanking-genes, genetic drift, epigenetics and environment have been widely discussed in literature [27–29]. Genomic aberrations have been identified in over 325 pluripotent stem cells from different mouse strains [30]. DNA double strand breaks like those that are induced during generation of new mouse models via homologous recombination occur also naturally at recombination hotspots where most meiotic recombination events cluster. Copy number variations have been shown to occur in cultivated mouse stem cells [31] and unexpected genomic rearrangements are a known yet underappreciated issue in generation of transgenic animals. Recently, analysis of the 8012 transgenic alleles in the Mouse Genome Database revealed that of only 5.2 % the exact chromosomal location is annotated and many of these lines display structural variations and other mutations [10].

We here describe occurrence of an unexpected tandem duplication during generation of a new *Agmo* mutant mouse strain by homologous recombination of a transgenic cassette. Such a duplication event has not been described so far. However, it might have also remained undetected during generation of other mouse strains due to the technical pitfalls that we discuss in the present manuscript.

The precise mechanism that might have induced the observed structural change remains speculative. The 5’ breakpoint of the duplication is located in an L1 LINE (chr12:37,204,420–37,210,460). These transposon elements make up ≈ 20 % of the mouse genome and the observed element has almost perfect matches (> 98 % identity) on every chromosome of the mouse. 48 copies with > 98 % identity were found distributed throughout chromosome 12 alone. No such L1 LINE was, however, found at the 3’ breakpoint at chr12:37,300,424, excluding a classical unequal crossing over as this requires a long homologous region [32]. The homology between the breakpoint regions was low and only some short homologies (2-8 bp) are present in the sequence context of the breakpoints. We may speculate that these short homologous tracts close to the breakpoints may have induced a microhomology-mediated recombination (comprehensively reviewed in [32]) but their significance remains elusive. Yan et al. described microhomology-mediated mechanisms as a prominent cause of random DNA integration in 36 screened transgenic mice [33] and a random integration of an apolipoprotein E transgene into the *Agmo* locus has been reported also by others, albeit without providing details [26]. In a general perspective the observation of the occurrence of a structural aberration with only low homology between the breakpoints highlight the importance of a careful genomic validation of transgenic models.

However, the presence of a segment of *Agmo* wild-type allele did not interfere with successful functional knockout of AGMO activity, making our original genotyping efforts by PCR misleading. Indeed, RT-PCR of mRNAs in transgenic *Agmo*-lacZ mice did not find evidence of mRNA molecules incorporating additional exons originating from the wild-type allele. This may indicate that, if present, such aberrant pre-mRNA might be retained in the nucleus for nonsense-mediated mRNA degradation. Besides enzymatic activities that perfectly matched the corresponding genotypes we also analyzed expression of the endogenous *Agmo* gene by RT-qPCR. This showed the expected decrease of *Agmo* mRNA in several organs of transgenic animals as compared to the wild-type littermate controls. We also found a significant correlation of *Agmo* gene expression and AGMO activity in all tested organs indicating that the level of enzymatic activity is directly regulated by the amount of mRNA.

Assessment of standard physiological parameters such as bodyweight, plasma lipids and fat distribution did not reveal any significant changes between homozygous knockout mice and their wild-type litter mates. The enzymatic activity of AGMO relies on BH4 as essential co-factor and knock-in mice with decreased BH4 levels demonstrate abnormal body fat distribution and increased blood cholesterol levels [34]. This effect was not mimicked by AGMO knockout in our mice, as shown by MRI and blood cholesterol/triglyceride analysis. Therefore, it can be assumed to be a mechanism independent of decreased ether lipid degradation by AGMO. Although AGMO has been implicated in a variety of human pathologies such as type 2 diabetes [20] and leishmania infection [15] it seems that from an unchallenged perspective *Agmo* knockout mice show no obvious phenotype. The influence on energy homeostasis and the susceptibility to infections of *Agmo* transgenic mice remains to be investigated in the future.

## Conclusions

The case reported here serves as an example for the pitfalls of common routine genotyping approaches.

While qPCR was eventually able to assign the correct genotype, only TLA and nanopore sequencing finally allowed fully resolving the precise nature of the genomic lesion induced during the recombination event. Especially nanopore sequencing is arising as promising and highly accessible technology for rapid profiling of genomic rearrangements and has been shown to significantly ease mapping of structural variation (SV) [35, 36]. For example, it has been used to map transgene integration sites and quantify transgene copies [37, 38], two challenging tasks when profiling transgenic animals. Together nanopore sequencing and TLA now allow detecting and mapping more complex genome lesions in a cost-convenient matter also in mammals. Indeed, 8 nanopore flow cells were required in a recent study to achieve 1.8X mouse genome coverage and map a 50 kb transgene array [37]. Conversely, in our study one flow cell was now sufficient to achieve > 4-fold coverage and map a > 95 kb large duplication. In light of the mounting evidence of unforeseen variations induced by homologous recombination [39] genomic validation of animal models is thus getting within reach and may become a standard procedure in the near future.

### Methods

### Generation of Agmo^tm1a(EUCOMM)wtsi^ knockout‐first mice and breeding

To generate *Agmo* knockout-first (*Agmo*-lacZ) mice, embryonic stem (ES) cell clone EPD0354_2_F05 (ES cell background JM8A3.N1 with Agouti coat color, allele: conditional knock-out first *Agmo* allele Agmo^tm1a(EUCOMM)Wtsi^, European Mouse Mutant Cell Repository (EuMMCR, Munich, Germany) were injected into C57BL/6 J blastocysts and transferred to pseudopregnant CD1 foster mothers. 156 embryos were implanted, from these, 30 pups were born (19.2 %) and all of them could be weaned (100 %). 6 male chimeric animals with the highest Agouti color contribution were bred with C57BL/6 J wild-type females and in the litters three heterozygous animals were identified. Breeding and animal experimentation of all mouse lines was approved by the Austrian Ministry of Education, Science, and Research (breeding: BMWFW-66.011/0094-WF/V/3b/2016 and BMWFW-66.011/0020-V/3b/2019; animal experimentation: BMWFW-66.011/0095-WF/V/3b/2016 and BMBWF-66.011/0019-V/3b/2019) and the UK Home Office Licenses 30/3080 and 30/2966. All *Agmo* mouse lines, FlpE deleter (B6.Tg(ROSA)26-FLP/Uhg) and CMV-cre deleter mice (Tg(CMV-cre)1Cgn) [40] were maintained on a C57BL/6 J background and were housed in individual ventilated cages with nesting material, in a 12 h/12 h light/dark cycle with standard diet (Ssniff Spezialdiäten GmbH, Soest, Germany; complete feed for rats and mice V1534-300, autoclaved) and water provided ad libitum.

### Numbering of positions on chromosome 12

All numbers of chromosomal positions refer to assembly GRCm38.p6 on chromosome 12 (accession number NC_000078.6).

### Fluorescent in situ hybridization (FISH)

For FISH experiments, a BAC probe specific for the *Agmo* locus on chromosome 12 (BAC RP24-270A18, chr12:37,225,445 − 37,407,098) labelled in red and a probe for the cassette (position 17,066 − 22,047 of the transgenic cassette, generated by PCR) labelled in green were hybridized onto metaphase spreads of fibroblasts isolated from wild-type and heterozygous *Agmo*-lacZ mice. For Fiber-FISH experiments the probes for the cassette, labelled in red, and the BAC probe for *Agmo* labelled in green, were hybridized to chromatin fibers of wild-type and heterozygous *Agmo*-lacZ fibroblasts.

### Genotyping, long‐range PCR and RNA preparation for qPCR

For conventional PCR, genomic DNA was extracted from ear notches and was analyzed by Taq DNA polymerase (Thermo Fisher Scientific Inc). For genotyping qPCR, the Monarch® Genomic DNA Purification Kit (New England Biolabs, Frankfurt am Main, Germany) and SsoFast EvaGreen Supermix (Bio-Rad Laboratories Inc., Hercules, USA) was used. For long-range PCR, we used LongAmp® Taq DNA polymerase (New England Biolabs). RNAs were extracted using the Monarch® Total RNA Miniprep Kit (New England Biolabs) and reverse transcription was done using 500 ng RNA and M-MLV Reverse Transcriptase, RNase H Minus, Point Mutant (Promega, Walldorf, Germany), recombinant RNAsin® Ribonuclease Inhibitor (Promega), dNTPs (Promega) and either oligo(dT)_15_ primer (Promega) to check for correct splicing or random hexamer primers (Microsynth, Balgach, CH) for RT-qPCR of *Agmo* gene expression using TaqMan assay technology and Luna® Universal Probe qPCR Master Mix (New England Biolabs). For primers see Additional file 3: Material and Methods. For a schematic representation of genotyping primer positions see Additional file 1: Fig. S1. All experimentally used mice were re-genotyped after sacrificing.

### Phenotyping methods

Weight measurement was conducted with standard scales. Weekly weight recordings of weaned mice were performed in parallel with every cage transfer.

Magnetic resonance imaging (MRI) was performed on a standard clinical 3 Tesla whole body scanner (Magnetom Skyra, Siemens, Germany) with a 45 mT/m gradient system. Anaesthetized mice were positioned prone within a combination of two small loop coils with an inner diameter of 3 cm (standard finger coils). For detailed information of analysis procedure see Additional file 3: Material and Methods.

Plasma lipid analysis: All animals were sedated with an intraperitoneal injection of ketamine (100 mg/kg body weight) (Animedica, Senden, Germany) and xylazine (10 mg/kg body weight) (Animedica). 500 µl of blood was withdrawn from de *Vena cava* using heparinized syringes and needles. Afterwards animals were sacrificed by cervical dislocation. Quantification of plasma cholesterol and triglycerides was performed by in vitro tests CHOL2 (Cobas, Vienna, Austria) and TRIGL (Cobas), respectively, for Roche automated clinical chemistry analyzers at the Central Institute of Medical and Chemical Laboratory Diagnostics, Medical University of Innsbruck.

### Isolation of embryos and MEFs from timed matings

For timed matings, 8 weeks old female mice were bred and checked for plugs every morning. At 12.5 d.p.f. female mice were sacrificed by cervical dislocation and embryos were removed and dissected. Embryonic heads were used for genotyping and lyzed in 450 µl DNA lysis buffer. The liver was removed and MEFs were prepared from the remaining body. Details are given in Additional file 3: Material and Methods.

### AGMO activity assay

Enzyme activity was measured as described in [41] with some modifications: Fatty aldehyde dehydrogenase was added in its recombinant form to the assay mixture and 1 mM dithioerythritol (Roth, Karlsruhe, Germany) final concentration in the assay mix was supplemented. We always analyzed tissues of littermates with different genotypes in parallel to exclude artefacts by day-to-day variability of the assay and variations in enzyme activities of different litters.

### Targeted locus amplification (TLA) in mouse splenocytes

Targeted Locus Amplification was performed by Cergentis B.V. (Utrecht, Netherlands) which provided also the protocol for isolation of murine splenocytes [26].

### Nanopore sequencing

Details about the sequencing and data analysis procedure are given in Additional file 3: Material and Methods. In brief, genomic DNA was isolated from homozygous *Agmo*-lacZ MEFs using the Qiagen EZ1 robot and two aliquots were sheared by needle shearing [42] to a range of 35–70 kb and 40-50 kb. Both samples were used for sequencing library preparation (Oxford Nanopore Technologies LSK-109 kit with minor modifications; Additional file 3: Material and Methods). The libraries were sequenced in three consecutive runs on an ONT MK1B MinION device (57.5 h total run time) with intermediate DNAse I washes (DNAse I, New England Biolabs). Data is available in the NCBI Sequence Read Archive at PRJNA667806.

### Sequencing data analysis

Basecalling was done with ONT Guppy v4.0.15 and quality assessed with NanoPlot v1.31.0 [43] (Additional file 1: Table S2). Reads were aligned to reference genome mm10 twice using minimap2 v2.17 [44] or NGMLR v0.2.7 [36] and processed using samtools v1.9. Structural variations (SV) were called on both alignments with sniffles v1.0.12 [36] and SVIM v1.4.1 [45]. Minimum two supporting reads were required to call an SV. SV calls were filtered for origin at GRCm38/mm10, chr12:37,200,000–37,299,999. Alignment of the breakpoint sequences chr12:37,206,133 and chr12:37,300,424 ± 30 bp were done using MAFFT [46] (allowing reverse complementation, --adjustdirectionaccurately parameter). Repeat elements are from the UCSC Genome browser RepeatMasker track [47, 48].

### Data presentation and statistics

Statistical analysis was performed by GraphPad Prism 7.03. Results are presented as mean ± S.E.M. unless otherwise indicated. The two-tailed unpaired Student’s t-test or two-way ANOVA followed by Tukey’s multiple comparisons post-hoc test was applied to test for significance. For nonparametric correlation Spearman’s rank was used and for Gaussian distributed data Pearson correlation was used.

## Supplementary Information

**Additional file 1.** Additional figures and tables.

**Additional file 2.** Additional fasta file.

**Additional file 3.** Additional materials and methods.

## Data Availability

Original sequencing data supporting the conclusions of this article are uploaded in the NCBI Sequence Read Archive (https://www.ncbi.nlm.nih.gov/sra) under Bioproject PRJNA667806.
