# Supplementary material for: When the genome bluffs: a tandem duplication event during generation of a novel Agmo knockout mouse model fools routine genotyping
Source: Cell Biosci. 2021 Mar 16;11:54. doi: 10.1186/s13578-021-00566-9 (PMC7962373; doi:10.1186/s13578-021-00566-9)
Supplement: Supplementary file 1 — Additional file 1. Additional figures and tables. [file 13578_2021_566_MOESM1_ESM.docx]

**Supplemental Data**

| ***tg/wt***  ***X***  ***tg/wt*** | *Agmo*-lacZ  **wt/wt** | *Agmo*-lacZ  **tg/wt** | *Agmo*-lacZ  **tg/tg** | ***total*** |
| --- | --- | --- | --- | --- |
|  | n =26 (29 %) | n = 42 (46 %) | n = 23 (25 %) | n = 91 |
| ***fl/wt***  ***X***  ***fl/wt*** | *Agmo*-flox  **wt/wt** | *Agmo*-flox  **fl/wt** | *Agmo*-flox  **fl/fl** | ***total*** |
|  | n = 20 (22 %) | n = 49 (53 %) | n = 23 (25 %) | n = 92 |
| ***fl/del***  ***X***  ***fl/del*** | *Agmo*-∆exon2  **fl/fl** | *Agmo*-∆exon2  **fl/del** | *Agmo*-∆exon2  **del/del** | ***total*** |
|  | n = 18 (22.5 %) | n = 40 (50 %) | n = 22 (27.5 %) | n = 80 |

**Supplemental Table S1. Mendelian distribution of litters from heterozygous breeding pairs of *Agmo*-lacZ, *Agmo*-flox and *Agmo*-∆exon2 mouse strains indicating no embryonic lethality of all mutant *Agmo* mouse strains.**

| **Sequencing run** | **Output [bp]** | **Median quality score [phred]** | **reads [n]** | **N50 [bp]** | **Median length [bp]** |
| --- | --- | --- | --- | --- | --- |
| # 1 | 4,794,698,218 | 11.5 | 237,107 | 43,382 | 10,324.0 |
| # 2 | 5,961,585,508 | 11.3 | 328,652 | 39,288 | 8,957.5 |
| # 3 | 2,332,429,145 | 11.0 | 133,584 | 39,026 | 8,236.0 |
| all | 13,088,712,871 | 11.3 | 699,343 | 40,669 | 9,223.0 |

**Supplemental Table S2. Sequencing quality figures.**


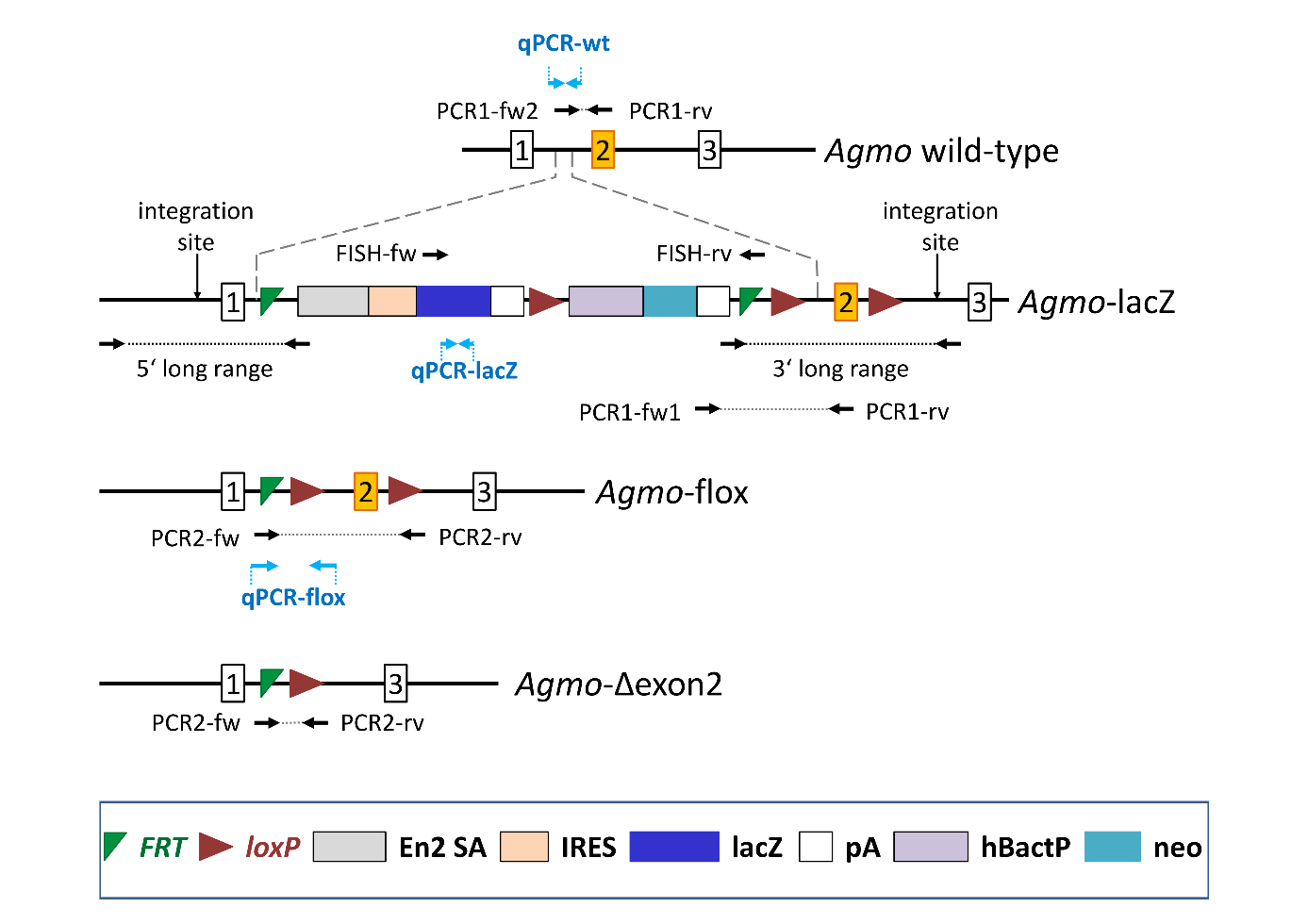


**Supplemental Figure S1. Schematic drawing for validation and genotyping of transgenic *Agmo* mouse strains.** Topology of (i) *Agmo* wild-type, (ii) *Agmo*-lacZ, (iii) *Agmo*-flox and (iv) *Agmo*-∆exon2 alleles and the position of the corresponding genotyping primers is shown. The critical exon 2 is marked in orange. PCR1 indicates the primers used for conventional genotyping against the transgenic cassette in *Agmo* knockout first mice. In blue the primer positions for wild-type and lacZ qPCR genotyping are shown that replaced the conventional genotyping procedure. PCR2 corresponds to the conventional genotyping method used for (iv) *Agmo*-∆exon2 mouse strains. QPCR-flox is used for *Agmo*-flox mouse strains (in blue). Additionally, the positions for long range PCR and fluorescent *in situ* hybridization (FISH) on the (ii) *Agmo*-lacZ allele, used to confirm correct integration of the transgenic cassette, are shown. FRT: FLP recognition target; loxP: locus of X-over P1 for bacteriophage P1 site-specific recombination; En2 SA: splice acceptor; IRES: internal ribosomal entry site; lacZ: coding sequence for β-galactosidase; pA: polyadenylation sequence; hBactP: human β-actin promoter; neo: neomycin resistance gene.


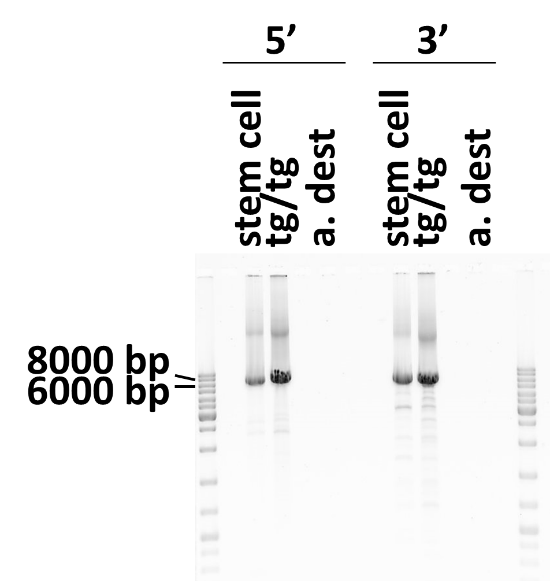


**Supplemental Figure S2. Long range PCR over the 5’ and 3’ homologous recombination arms in the parental EUCOMM stem cell clone and a homozygous *Agmo*-lacZ mouse.** Correct integration of the knockout-first construct was checked in the stem cell clone F05 from EUCOMM used for blastocyst injection and a homozygous (tg/tg) *Agmo*-lacZ mouse by PCR over the 5’ (product size: 7208 bp) and 3’ (product size: 6793 bp) homologous recombination arms (for primer sequences see Supplemental Material and Methods and Supplemental Figure S1).


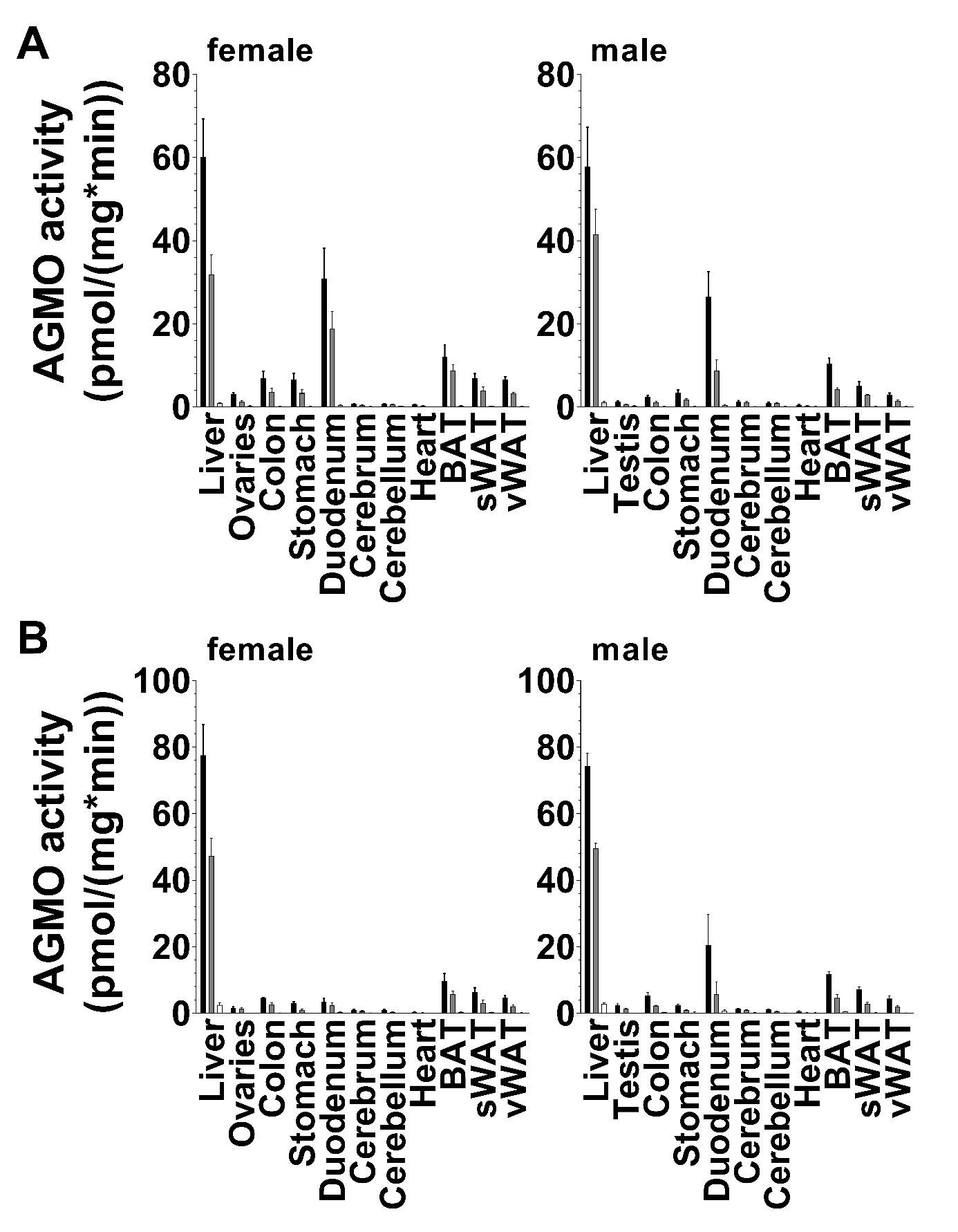


**Supplemental Figure S3. Impact of the genotype on AGMO enzymatic activity in 11 organs of male and female *AGMO* knockout mice.** AGMO activity was measured in all three genotypes of male and female (A) *Agmo*-lacZ mice (n = 6-13) and (B) *Agmo*-Δexon2 mice (n = 3-5) (genotypes determined by qPCR). Black bars represent wild-type, grey bars heterozygous and white bars homozygous mice. Mean ± S.E.M.


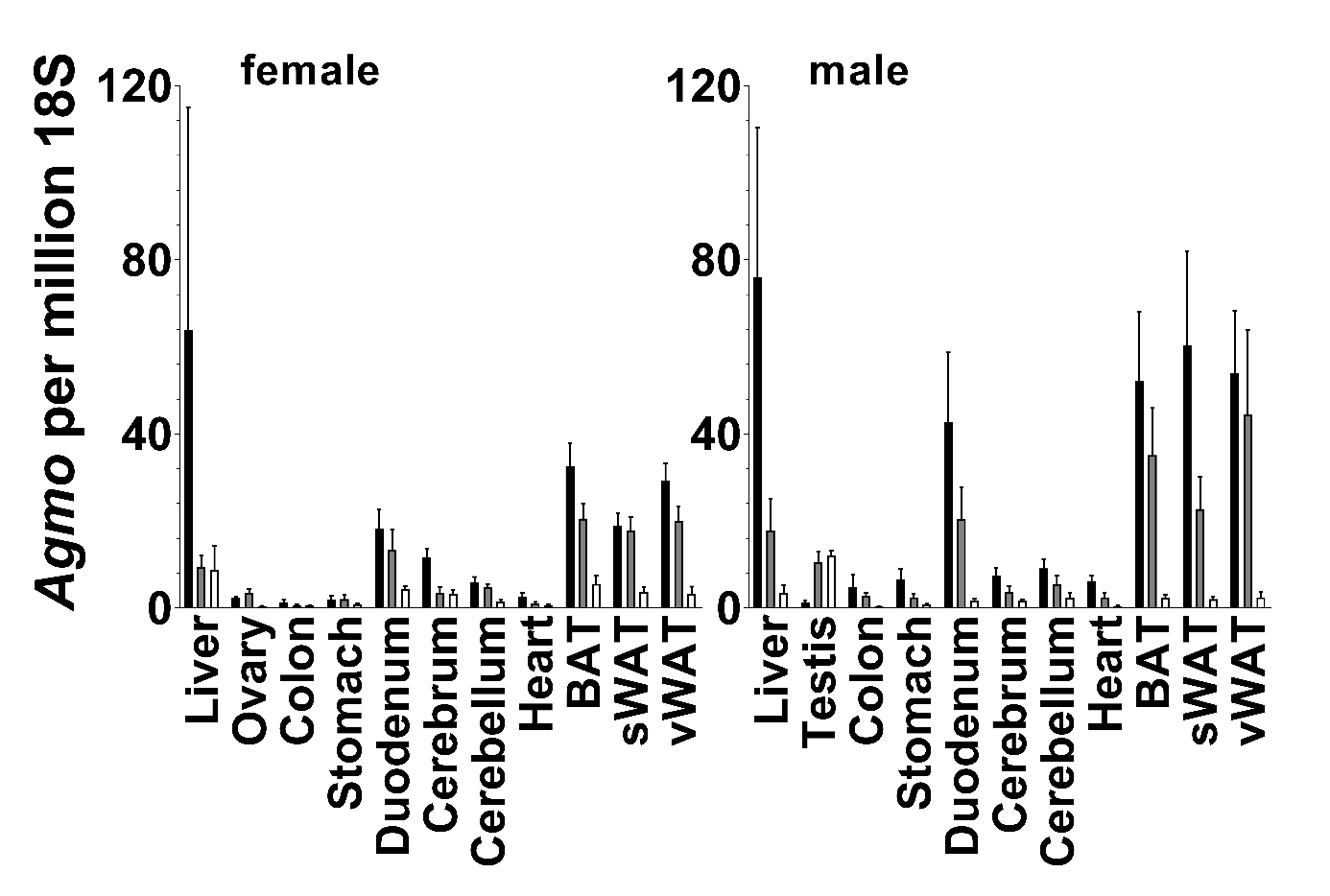


**Supplemental Figure S4. Impact of the genotype on *Agmo* gene expression in 11 tissues of male and female *Agmo*-lacZ mice.** Gene expression of reverse transcribed RNA was analyzed by qPCR as detailed in Supplemental Material and Methods in all three genotypes of both, male and female mice (n = 5-10). Black bars represent wild-type, grey bars heterozygous and white bars homozygous mice. Mean ± S.E.M.


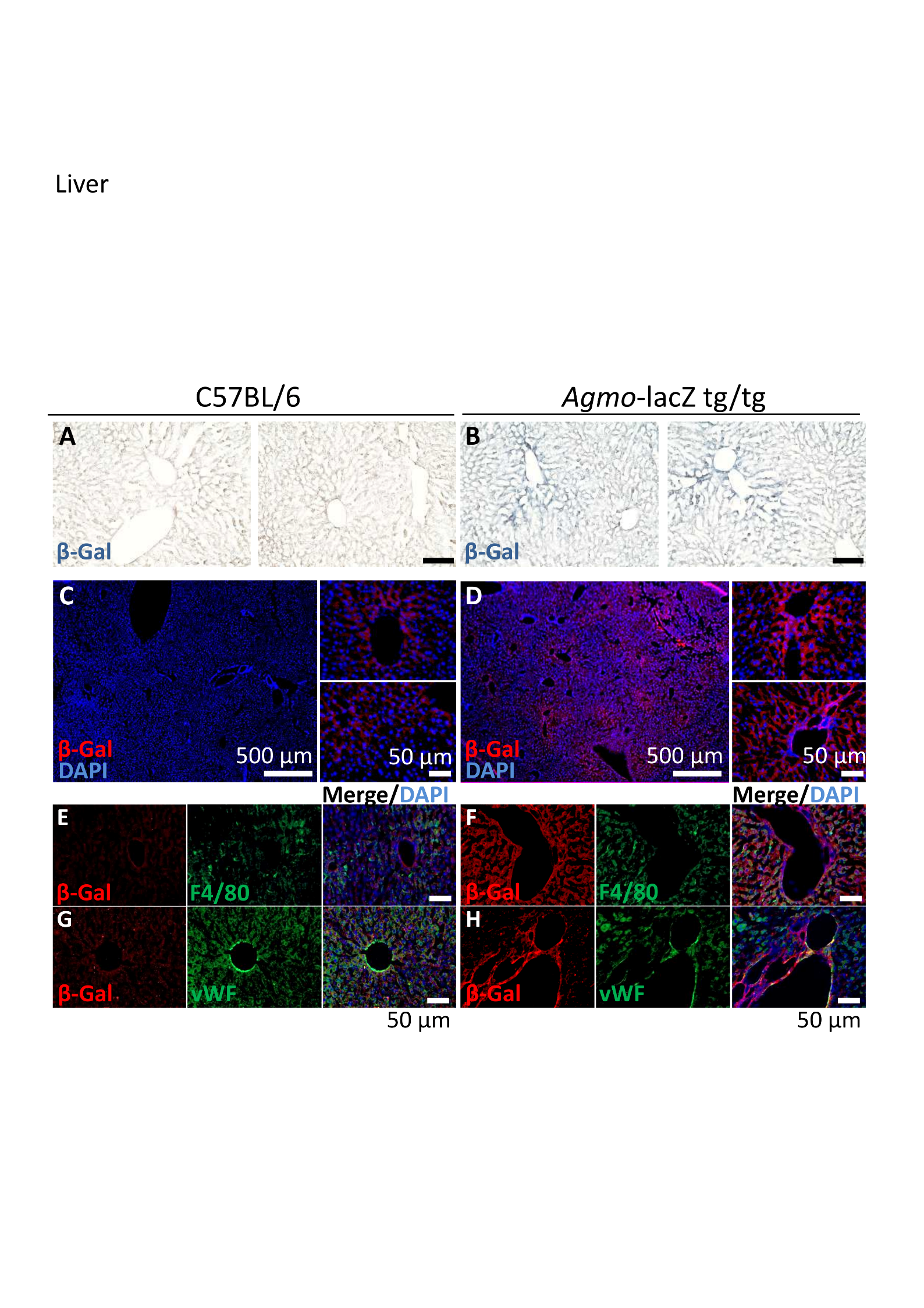


## Supplemental Figure S5. Histological sections of *Agmo*-lacZ transgenic reporter mice and controls showing localization of AGMO in the liver. β-galactosidase staining of wild-type (A) and *Agmo*-lacZ (B) liver sections. Overview of immunohistochemistry staining using a fluorescent antibody against β-galactosidase (red) in wild-type (C) and homozygous *Agmo*-lacZ (D) mice. Antibody staining with F4/80 (marker for eosinophils; green), von Willebrand Factor (vWF; green) and β-galactosidase (red) in lacZ transgenic animals (D, F and H) the corresponding wild-type control animals are shown in (C, E and G). Counterstaining is DAPI (blue). n = 3

*
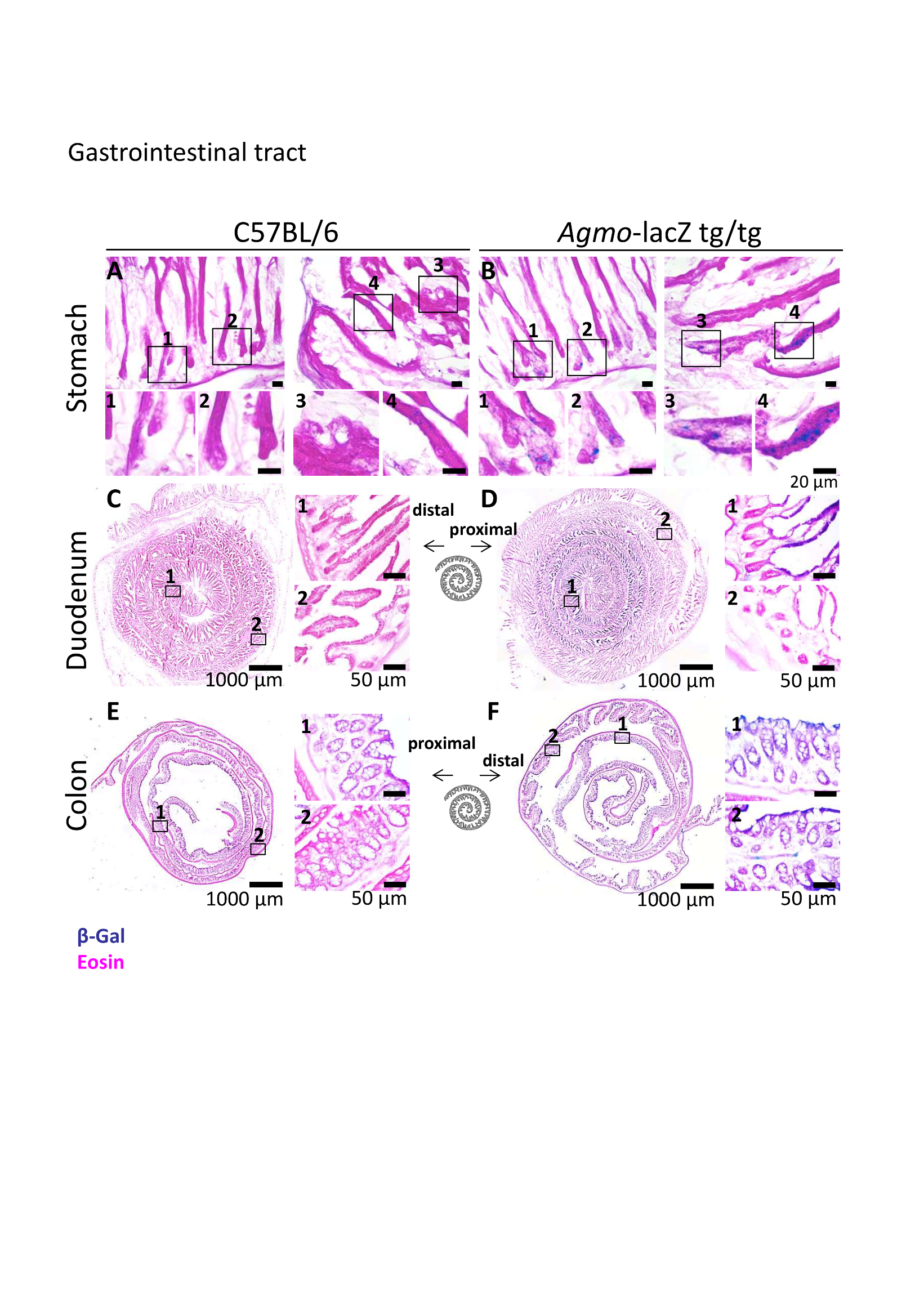
*

**Supplemental Figure S6. β-galactosidase staining of the gastrointestinal tract of homozygous *Agmo*-lacZ and wild-type control mice is only present in homozygous *Agmo*-lacZ animals.** Histological sections of the stomach in wild-type (A) and *Agmo*-lacZ (B) stained with β-galactosidase. β-galactosidase staining in duodenum of wild-type (C) and homozygous *Agmo*-lacZ mice (D). β-galactosidase staining in colon tissue sections of homozygous *Agmo*-lacZ (F) compared to wild-type controls (E). Eosin was used to stain the cytoplasm and extracellular matrix. n = 3


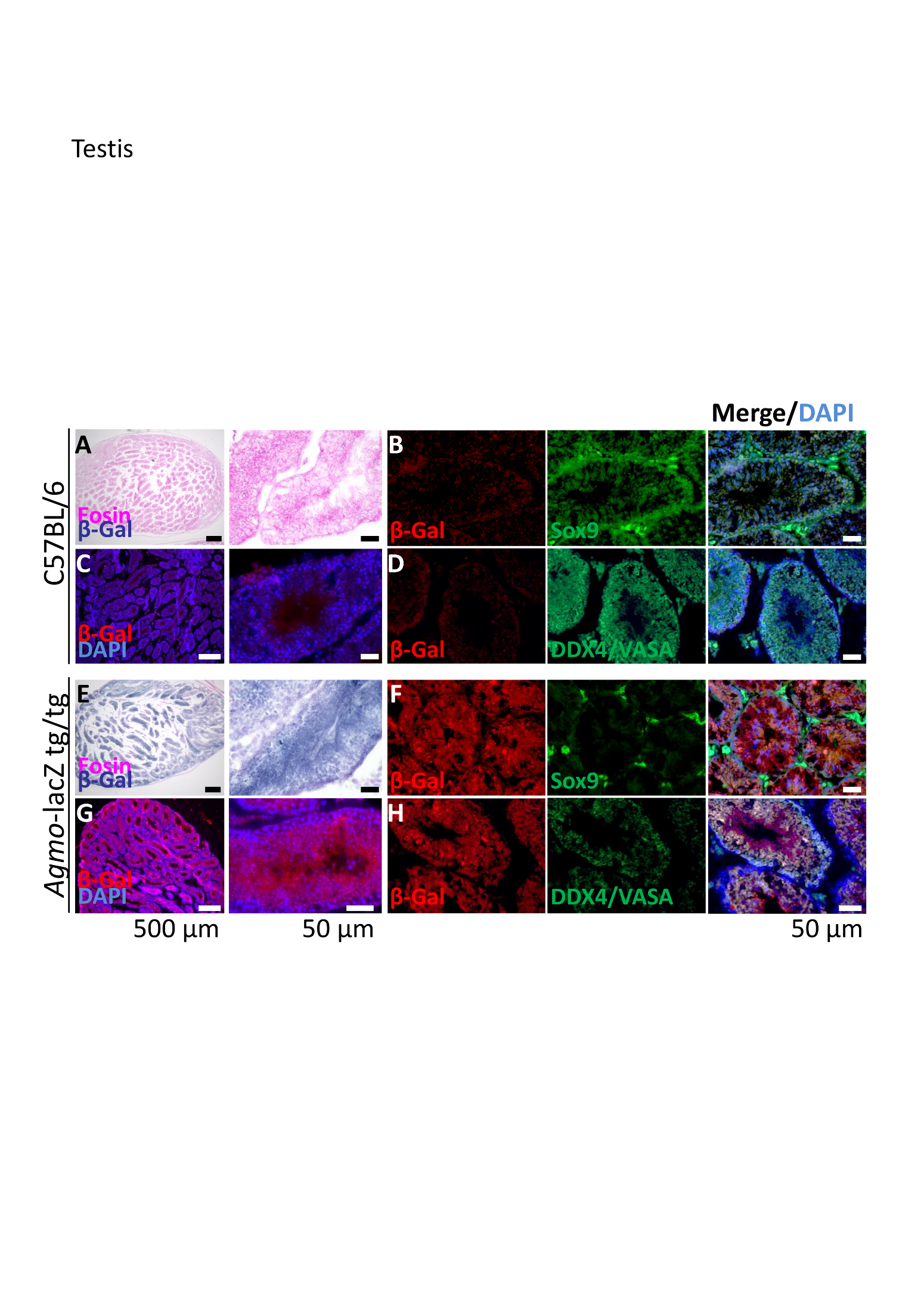


**Supplemental Figure S7. β-galactosidase staining and immunofluorescence of testicular tissue sections showing localization of AGMO in testicular germ cells.** β-galactosidase and eosin staining of wild-type (A) and *Agmo*-lacZ mice (E). Immunofluorescence pictures of tissue sections incubated with β-galactosidase antibody (red), DDX4/VASA antibody (green) in testicular germ cells (H) and Sox9 antibody (Sertoli cell marker; green) (F) of *Agmo* knockout animals. The corresponding wild-type controls are shown in (B) for counterstaining with β-galactosidase (red) and Sox9 (green) and (D) counterstaining with β-galactosidase (red) and DDX4/VASA (green). Counterstaining is DAPI (blue). n = 3


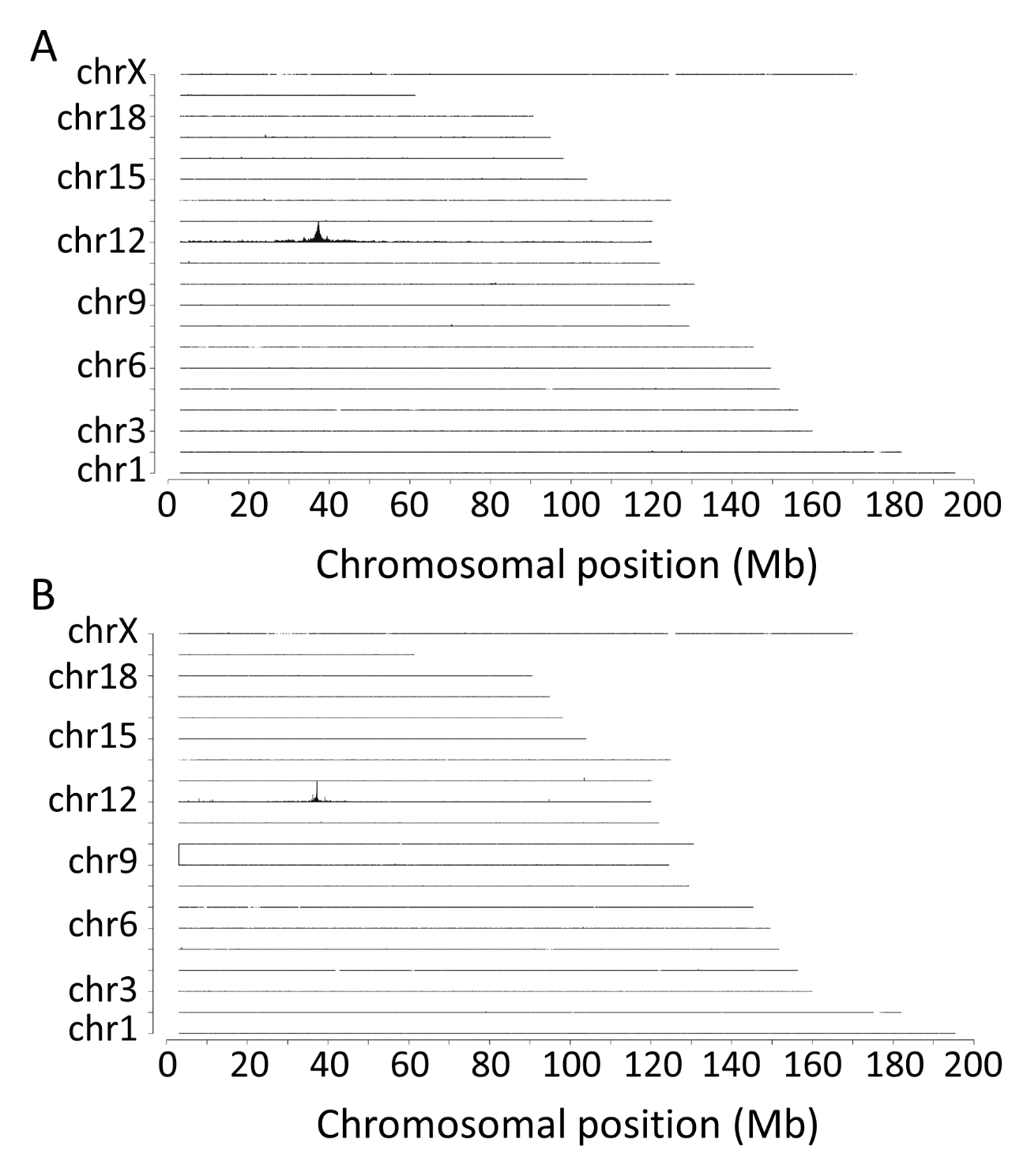


**Supplemental Figure S8. TLA results for whole genome** **showing integration at the expected *Agmo* genome locus at approximately chr12:37,200,000-37,300,000.** Whole genome TLA result for the mutant cassette (A) and the wild-type cassette (B). Supplemental Figure S8 maps the *Agmo* locus. A second, sharply delimited peak at chromosome 9qA1 (chr9:3,000,000-3,050,000) corresponds to an unspecific coverage peak in a large DNA satellite segment with reads being aligned there despite low mapping quality and many mismatches.


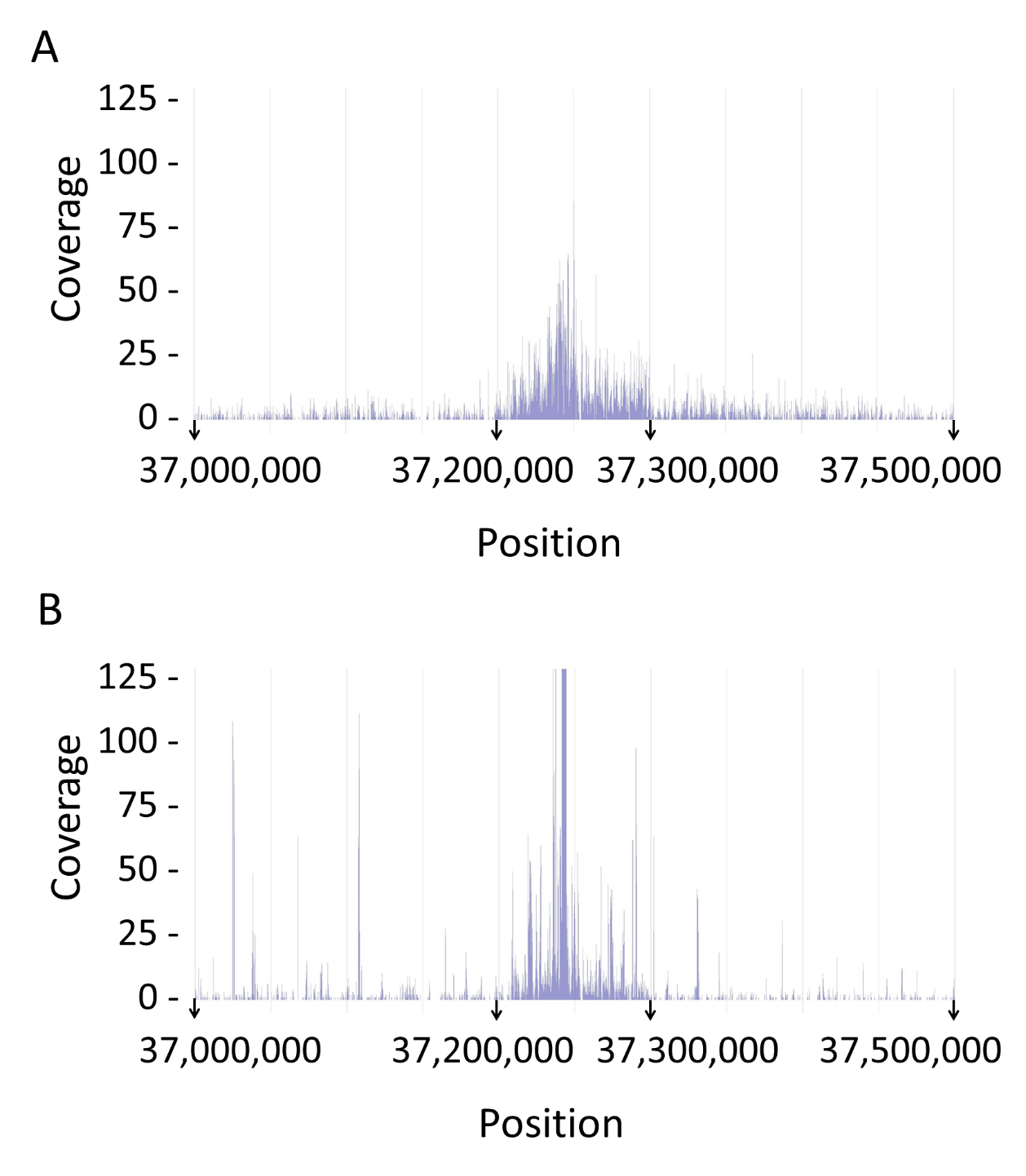


**Supplemental Figure S9. TLA results for the *Agmo* locus show integration at the same locus for the wild-type cassette and the mutant cassette.** TLA result zoomed in on the *Agmo* locus chr12:37,000,000-37,500,000 for the mutant cassette (A) and the wild-type (B).

**
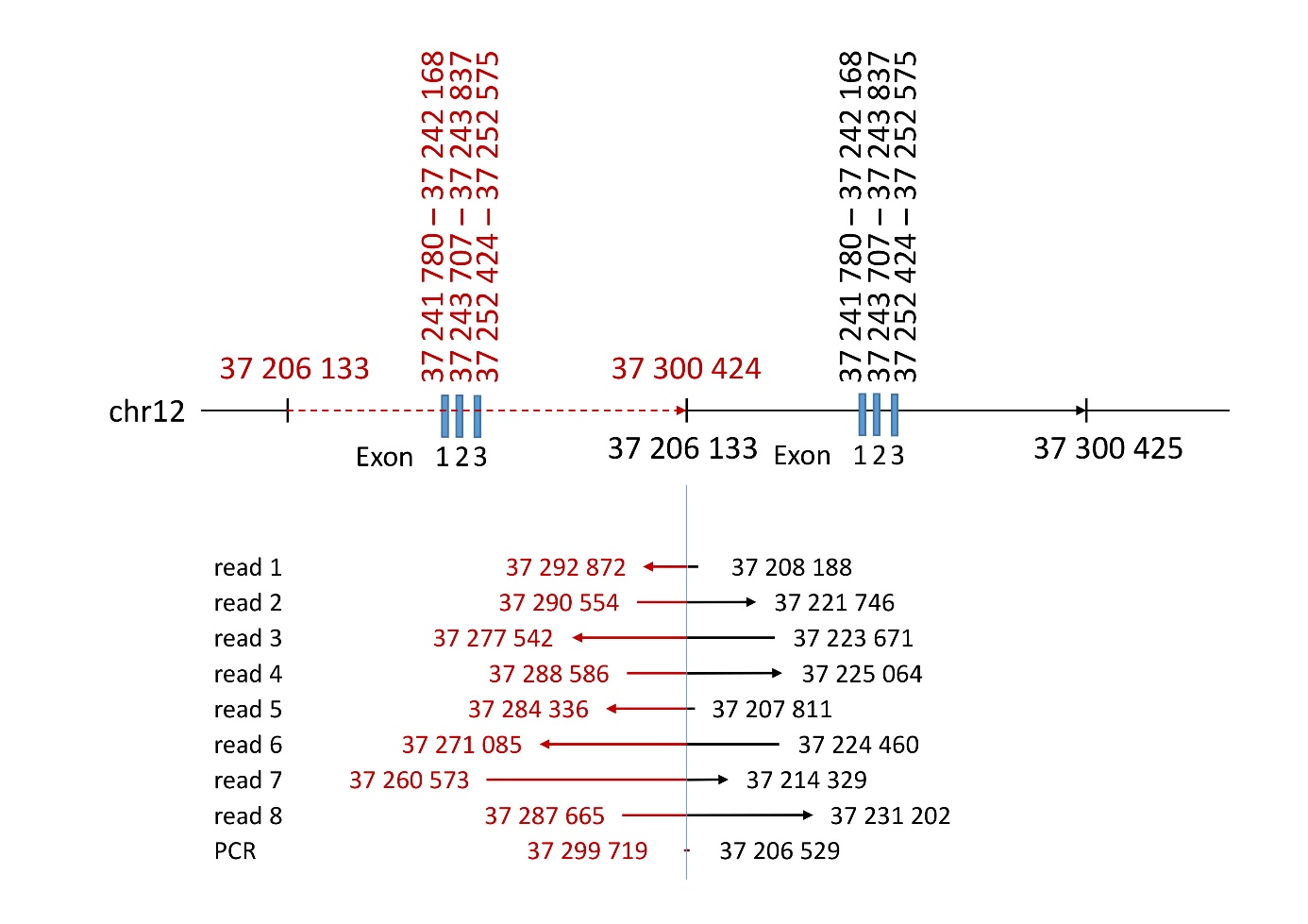
**

**Supplemental Figure S10: Confirmation of the tandem duplication of the *Agmo* locus in the homozygous *Agmo*-lacZ mouse on chromosome 12 by nanopore sequencing reads and by PCR.** All eight reads from nanopore sequencing and the PCR reaction align on chromosome 12 within the *Agmo* locus where the transgenic cassette had been introduced through homologous recombination. These reads where concordantly identified as supportive reads by all algorithm combinations. The sequence of these reads as well as of sequenced PCR products is reported in the Supplemental Fasta file.


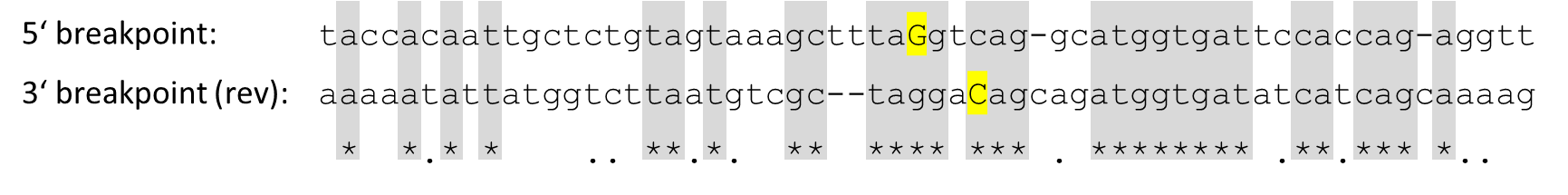


**Supplemental Figure S11: Alignment of the 5’ breakpoint with the reverse complement of the 3’ breakpoint (±30 bp each) shows low homology around the breakpoints with some short local homologies in the context sequence.** Alignment was done using MAFFT and allowing reverse complementation of sequences to all possible combinations. Other combinations did not show appreciable homology levels. Breakpoints are shown in yellow and upper case.
