# Supplementary material for: When the genome bluffs: a tandem duplication event during generation of a novel Agmo knockout mouse model fools routine genotyping
Source: Cell Biosci. 2021 Mar 16;11:54. doi: 10.1186/s13578-021-00566-9 (PMC7962373; doi:10.1186/s13578-021-00566-9)
Supplement: Supplementary file 3 — Additional file 3. Additional materials and methods. [file 13578_2021_566_MOESM3_ESM.docx]

**Supplemental Material and Methods**

**PCR and primer sequences**

For an overview of position of primers see Supplemental Fig. S1

FISH experiments: 5’-CAAACTGGCAGATGCACGGTTACGATG-3’ (fw) and 5’-GAGAATAGGAACTTCGGAATAGGAACTTCTC-3’ (rv). Long-range PCR to validate correct integration of the cassette: 5’-GATGACAGTAACGTGGTACATAAG-3’ (fw) and 5’-CACAACGGGTTCTTCTGTTAGTCC-3’ (rv) against the 5’ arm of homology and 5’-CTATGAAAGGTTGGGCTTCGGAATC-3’ (fw) and 5’-ACAGCGTCACCTCTCTGAACAAAAG-3’ (rv) against the 3’ arm of homology.

Conventional PCR for genotyping of the transgenic cassette (PCR1 in Supplemental Fig. S1): 5’-TCGCCTTCTTGACGAGTTCTTCTG-3’ (fw1), 5’-TTTGAAGGGATTCTCCTAGAGTGAC-3’ (fw2) and 5’-TAGATGTGAGAGCATCATCCAAACG-3’ (rv) (band size: wt = 476 bp and transgene = 1090 bp). Conventional PCR for genotyping of Ubi-cre deleter mice: 5’-CTGACCGTACACCAAAATTTGCCTG-3’ (fw) and 5’-GTTCTGCCAATATGGATTAACATTC-3’ (rv) (band size: wt = no band and positive mice = 600 bp). Conventional PCR for genotyping of *Agmo*-∆exon2 mice (PCR2 in Supplemental Fig. S1) band size: wt 867 bp and transgene without exon 2 = 126 bp): 5’-CGCCGGAACCGAAGTTCCTA-3’ (fw) and 5’-TGAGCATGAACTGATGGCGA-3’ (rv).

QPCR primers for wild-type (qPCR-wt): 5’-TAGTTCTAGACTGCCTCATTTG-3’ (fw) and 5’-AACCTATCTTTTGACCCTGAAC-3’ (rv). QPCR primers for *Agmo*-lacZ (qPCR-lacZ): 5’-TCTGTATGAACGGTCTGGTC-3’ (fw) and 5’-TATTCGCTGGTCACTTCGAT-3’ (rv). QPCR primers for *Agmo* floxed allele (qPCR-flox): 5’-GCCGGAACCGAAGTTCCTA -3’ (fw) and 5’-TTGTACAAGAAAGCTGGGTCT -3’ (rv). QPCR primers for the reference gene *Eef2*: 5’-AGGCCTGTGTAATATAGCTGCG-3’ (fw) and 5’-CTCTGTGTAGTTTGTAGCTCTGTCT-3’.

Primers used to confirm the duplication event: 5’-AGTCTCTGTATGGTCCTTCCTTTC-3’ (fw) and 5’-CTCAAGCAACTCAATGGCCTTTCTC-3’ (rv) (band size = 1127 bp).

Primers used to determine whether correct splicing occurred: 5’-AACAGAAATGAGGAGCCCAGGAG-3’ (fw) and 5’-GCTTTTTCCAAGAGCCAGAGGTG-3’ (rv)

Primer sequences for *Agmo* gene expression studies by RT-qPCR were as follows: *18S*: 5’- CCATTCGAACGTCTGCCCTAT-3’ (fw), 5’-TCACCCGTGGTCACCATG-3’ (rv), 5’-ACTTTCGATGGTAGTCGCCGTGCCT-3’ (probe); *Agmo*: 5’-CTTTCTTAGGAGTTGACTTTGGCTACT-3’ (fw), 5’-TGTGCTGCCCAGAAAATATTAATC-3’ (rv), 5’-CTGGTTCCACCGCATGGCTCATG-3’ (probe).

For conventional PCR, genomic DNA was extracted from ear notches with DNA lysis buffer (200 mM NaCl, 100 mM Tris HCl pH 8.5, 5 mM EDTA pH 8.0, 0.2% SDS; proteinase K (Thermo Fisher Scientific Inc, Waltham, USA) added freshly in a ratio of 1:300), was incubated over night at 55°C and amplified using Taq DNA polymerase (Thermo Fisher Scientific Inc).

For genotyping by qPCR, genomic DNA was prepared by Monarch® Genomic DNA Purification Kit (New England Biolabs, Frankfurt am Main, Germany) and a total amount of 20 ng gDNA was analyzed with the SsoFast EvaGreen Supermix (Bio-Rad Laboratories Inc., Hercules, USA).

To verify the duplication event Taq DNA polymerase (Thermo Scientific Inc) was used.

For long-range PCR, we used LongAmp® Taq DNA polymerase (New England Biolabs).

RNAs were extracted using the Monarch® Total RNA Miniprep Kit (New England Biolabs) and reverse transcription was done using 500 ng RNA and M-MLV Reverse Transcriptase, RNase H Minus, Point Mutant (Promega, Walldorf, Germany), recombinant RNAsin® Ribonuclease Inhibitor (Promega), dNTPs (Promega) and either oligo(dT)_15_ primers (Promega) for RT-PCR or random hexamer primers (Microsynth, Balgach, CH) for RT-qPCR.

For PCR determining correct splicing of the *Agmo* mRNA Taq DNA Polymerase (Thermo Scientific), 10 µM dNTP mix (Promega) and DMSO (Stratagene, La Jolla, California, USA) was used.

For RT-qPCR experiments analyzing *Agmo* gene expression in 11 tissues the TaqMan assay technology using Luna® Universal Probe qPCR Master Mix (New England Biolabs) and the Mx3005P qPCR system (Agilent, Santa Clara, CA, USA) were used. Taqman probes were labelled with FAM (5’) and TAMRA (3’).

To visualize bands on agarose gels SYBR® safe DNA Gel Stain (Invitrogen, Carlsbad, USA) was used. **Cloning of PCR products and purification for sequencing**

For ligation of PCR products, the TOPO™ TA Cloning Kit (Invitrogen) and One Shot™ TOP10 competent *E. coli* were used for transformation and plated on LB-agar plates containing 250 µg/ml Ampicillin. Bacterial clones were isolated and further grown in suspension overnight in Terrific-Broth-Medium supplemented with 250 µg/ml Ampicillin (Roth, Karlsruhe, Germany). Plasmids were isolated using the QIAprep Spin Miniprep Kit (Qiagen, Hilden, Germany) and sent for sequencing to Microsynth.

**Histology and tissue immunofluorescence**

Terminally anesthetized 8-12 weeks old *Agmo*-lacZ tg/tg mice were perfused transcardially with 0.9% saline followed by 2% paraformaldehyde (PFA). Tissues were excised and post-fixed in 2% PFA for 0.5-2 h followed by cryoprotection in 10, 20 and 30% sucrose at 4°C and subsequently embedded in Tissue-Tek® O.C.T. Compound (Science Services, Munich, Germany). 12 µm sections were cut on a cryotome, air-dried and stored at -80°C. Tissue slices of *Agmo*-lacZ wt/wt or C57BL/6 mice served as lacZ negative controls.

Tissue sections were treated with nitrotetrazolium blue chloride (NTB), which reacts with β-galactosidase to a dark blue to purple precipitate, visualizing lacZ positive cells. Therefore, cryosections were post-fixed for 5 min in 2% PFA, washed in 2 mM MgCl_2_/PBS and 3 times in detergent wash (0.1% sodium deoxycholate, 0.02% Nonidet P40 in 2 mM MgCl_2_/PBS, pH 7.5) for 5 min at room temperature. Tissue slices were covered with staining solution (0.5 mg/ml NTB, 5 µg/ml phenazine methosulfate (PMS) in detergent wash) and incubated for 3 to 24 h at 37°C depending on tissue background reaction and lacZ expression strength. Tissue slices of lacZ negative animals were always run in parallel. The reaction was stopped by washing the slides at least 3 times in PBS for 5-10 min. Counter-staining with eosin was performed, followed by dehydration in increasing ethanol concentration and xylene before embedding in Pertex mounting medium. For tissue immunofluorescence, slices were permeabilized in PBS with 0.1% Triton-X-100 (PBST), blocked with 3% BSA in PBS for 60-120 min at room temperature and subsequently incubated with primary antibodies in 1% BSA in PBS overnight at 4°C. Slices were then incubated with the respective secondary antibodies for 2 h at room temperature and nuclei were counterstained with DAPI. Images were taken with an inverted fluorescence microscope (Axiovert 200, Carl Zeiss, Jena, Germany) or a Keyence BZ-9000 fluorescence microscope in the bright-field or fluorescence mode.

The following primary antibodies were used for immunofluorescence: chicken α-beta-galactosidase (1:500 or 1:1,000; Abcam Cat# ab9361, RRID:AB_307210), rabbit α-von Willebrand Factor (1:250; Millipore Cat# AB7356, RRID:AB_92216), rabbit α-DDX4/VASA (1:100; Proteintech Cat# 51042-1-AP, RRID:AB_2092998), rabbit α-Sox9 (1:100; Cell Signaling Technology Cat# 82630, RRID:AB_2665492), rat α-F4/80 (1:200; Thermo Fisher Scientific Cat# 14-4801-82, RRID:AB_467558). Respective secondary antibodies used were as follows: Alexa fluor 555 α-chicken (1:800; Innovative Research Cat# A21437, RRID:AB_1500593), Alexa fluor 488 α-rabbit (1:800; Thermo Fisher Scientific Cat# A27034, RRID:AB_2536097) and Alexa fluor 488 α-rat (1:800; Thermo Fisher Scientific Cat# A-11006, RRID:AB_2534074).

**Magnetic Resonance Imaging (MRI)**

For fat quantification a T2-Weighted Dixon Turbo Spin Echo sequence (1) was used to acquire crossectional images through the mouse in coronal orientation, covering the whole mouse volume (TR = 1,600 ms, driven equilibrium pulse: on, TE = 75 ms, echo train length: 10, FOV: 196 x 172, acquisition matrix: 384 x 235, number of images: 17, slice thickness: 1.5 mm, spacing between slices 1.65 mm, voxel size: 0.51 mm x 0.51 mm x 1.5 mm, number of averages: 1, acquisition time: 4:21 min). During image reconstruction the sequence automatically generates T2-weighted fat- and water-separated images, which were then used to compute fat and body volumes. For this, image post processing was performed using ImageJ software (2). For volume calculation, fat was segmented semiautomatically based on the fat separated images using predefined thresholds. In contrast whole body volumes of the mice were calculated based on water-separated images, also using semiautomatic, threshold based segmentation. For the MRI acquisition, mice were sedated with an intraperitoneal injection of ketamine (100 mg/kg body weight) (stock: 100 mg/ml; Animedica, Senden, Germany), xylazine (10 mg/kg body weight) (stock: 20 mg/ml; Animedica). After the imaging animals were sacrificed by cervical dislocation.

**Isolation of mouse embryonic fibroblasts (MEFs)**

Embryos of timed matings were harvested at day E-12.5. The heads and livers were removed and embryonic bodies were washed in sterile 1x PBS supplemented with 1% Pen/Strep (Sigma, Vienna, Austria) and transferred to 1x trypsin-EDTA solution (Sigma) and incubated for 30 min in a 37°C water bath. The suspension was vortexed every 5-10 min. After digestion the suspension was further homogenized by pipetting up and down using a 1 ml pipet tip and moved to a 10 cm petri dish containing DMEM/GlutaMAX high glucose (Life Technologies, Vienna, Austria) supplemented with 10% FCS (Invitrogen) and 1% Pen/Strep (Sigma). Cells were grown in a humidified atmosphere with 5% CO_2_ at 37°C. After cells reached about 90% confluency, they were frozen in 90% FCS and 10% DMSO and aliquots were stored in liquid nitrogen. For nanopore sequencing, MEFs were harvested at a density of 800,000 cells in 250 µl PBS.

**Isolation of mouse splenocytes for TLA**

The isolated spleen was homogenized by gently pushing it through a 40 µm mesh (Fisher Scientific, Schwerte, Germany) and was then centrifuged at 250 x g at room temperature for 10 minutes in 10% FCS/1x PBS. The pellet was resuspended in 25 ml red blood cell lysis buffer (154 mM ammonium chloride, 10 mM potassium bicarbonate, 193 µM EDTA) and incubated for 5 minutes at room temperature. Afterwards the cell suspension was centrifuged at 250 x g for 10 minutes at room temperature and washed additionally with 10% FCS/1x PBS. A total number of > 5 x 10^7^ was stored at -80°C in 1 ml 10% FCS/10% DMSO/1x PBS.

**Nanopore Sequencing**

DNA was isolated from MEFs using the Qiagen EZ1 nucleic acid isolation robot. 4.5 µg DNA (54.9 µl) were sheared by 20 passes through a 26G needle (BD Microlance 3 26G x ¾ on Braun Injekt-F Solo syringe; AGMOSeq1 sample) and 3.5 µg (42.7 µl) were sheared using 13 passes through a 30G needle (BD Microlance 3, 30 G on Braun Injekt-F Solo syringe, AGMOSeq2 sample) following the indications of (3). Preliminary shearing experiments had indicated that these conditions generate DNA with the majority of fragments in the range of approximately 35-70 kb (26G) and 40-50 kb (30G).

3.68 µg of AGMOSeq1 and 2.38 µg of AGMOSeq2 were used for library preparation using the Oxford Nanopore Technologies (ONT) LSK-109 library prep kit. Library was done according to manufacturer protocol but prolonging the final library elution to 10 min at 37°C plus additional 10 minutes at room temperature. Library yields were 42.24 pmol for AGMOSeq1 (1.39 µg) and 41.72 pmol (1.24 µg) for AGMOSeq2 library. Quantifications were done using a LifeTechnologies Qubit 3 fluorometer with DNA HS kit. The two libraries were sequenced in three consecutive runs on a R9.4D flow cell on an ONT MK1B MinION device for a total of 57.5 h (run 1: 22 pmol AGMOSeq1 for 16 h; run 2: 27 + 13 pmol AGMOSeq2 for 16.5 h plus additional 9 hours after flow cell reloading; run 3: 20 pmol AGMOSeq1, 16 h). Manual DNAse I washes (DNAse I, New England Biolabs) were performed according to manufacturer protocol between the runs and before library reloading to free blocked pores.

**Supplemental References**

1. Ragan DK, Bankson JA. Two-point Dixon technique provides robust fat suppression for multi-mouse imaging. Journal of magnetic resonance imaging : JMRI. 2010;31(2):510-4.

2. Schneider CA, Rasband WS, Eliceiri KW. NIH Image to ImageJ: 25 years of image analysis. Nat Methods. 2012;9(7):671-5.

3. Tyson J. Rocky Mountain adventures in Genomic DNA sample preparation, ligation protocol optimisation / simplification and Ultra long read generation. 2020;dx.doi.org/10.17504/protocols.io.7euhjew.
